# Supplementary material for: Relative Importance of Deterministic and Stochastic Processes on Soil Microbial Community Assembly in Temperate Grasslands
Source: Microorganisms. 2021 Sep 10;9(9):1929. doi: 10.3390/microorganisms9091929 (PMC8469474; doi:10.3390/microorganisms9091929)
Supplement: Supplementary file 1 [file microorganisms-09-01929-s001.zip › microorganisms-1369598-supplementary/Supplementary Files.pdf]

Table S1 Comparison of dissimilarity of soil microbial communities between top- and sub−soils in temperate grasslands with different statistical approaches.

Domains	Datasets	Adonis		ANOSIM		MRPP	
		F	*p*	*R*	*p*	*δ*	*p*
**Bacteria**							
	Bray−Curtis	14.73	0.001	0.54	0.001	0.41	0.001
	weighted UniFrac	18.47	0.001	0.57	0.001	0.20	0.001
**Archaea**							
	Bray−Curtis	19.34	0.001	0.45	0.001	0.46	0.001
	weighted UniFrac	18.83	0.001	0.34	0.001	0.04	0.001

All three tests are non-parametric multivariate analyses based on Bray–Curtis and weighted UniFrac dissimilarities among samples, including the permutational multivariate analysis of variance (Adonis), analysis of similarity (ANOSIM) and multiple response permutation procedure (MRPP).

Table S2. First principal component (PC 1) extracted from the principal component analysis (PCA) for the five environmental groups and results of Kaise−Meyer−Olkin (KMO) test and Bartlett test of sphericity (BS) for the variables used for the principal component analysis (PCA) in the paper. The plus (+) and minus (−) in the parentheses refer to the positive or negative correlation between the PC1 and the individual variables.

**Pincomp**	**Parameters**	**PC 1 (%)**		**KMO values**		**BS test**	
Contemporary climate PC 1	MAP (+), MAT (−), Aridity index (+), SWC (+)	92.6		0.68		$\chi^2 = 292, p < 0.05$	
Aboveground vegetation PC 1	Plant species richness (+), aboveground biomass (+), NPP (+)	75.3		0.57		$\chi^2 = 45, p < 0.05$	
		**Top**	**Sub**	**Top**	**Sub**	**Top**	**Sub**
Soil fertility PC 1	Soil total carbon (+), soil organic carbon (+), soil total nitrogen (+), soil total phosphorus (+)	88.7	68.7	0.82	0.66	$\chi^2 =$ 206, $p <$ 0.05	$\chi^2 =$ 97, $p <$ 0.05
Soil mineral content PC 1	Soil silt (+), soil sand (−), soil extractable Fe (+), soil extractable Al (+)	74.5		0.69		$\chi^2 =$ 209, $p < 0.05$	
	Soil silt (+), soil sand (−), soil extractable Fe (+)		66.7		0.50		$\chi^2 =$ 221, $p < 0.05$

MAP, mean annual precipitation; MAT, mean annual temperature; SWC, soil water content; NPP, net primary productivity; Fe, iron; Al, aluminum; Top, topsoil; Sub, subsoil.

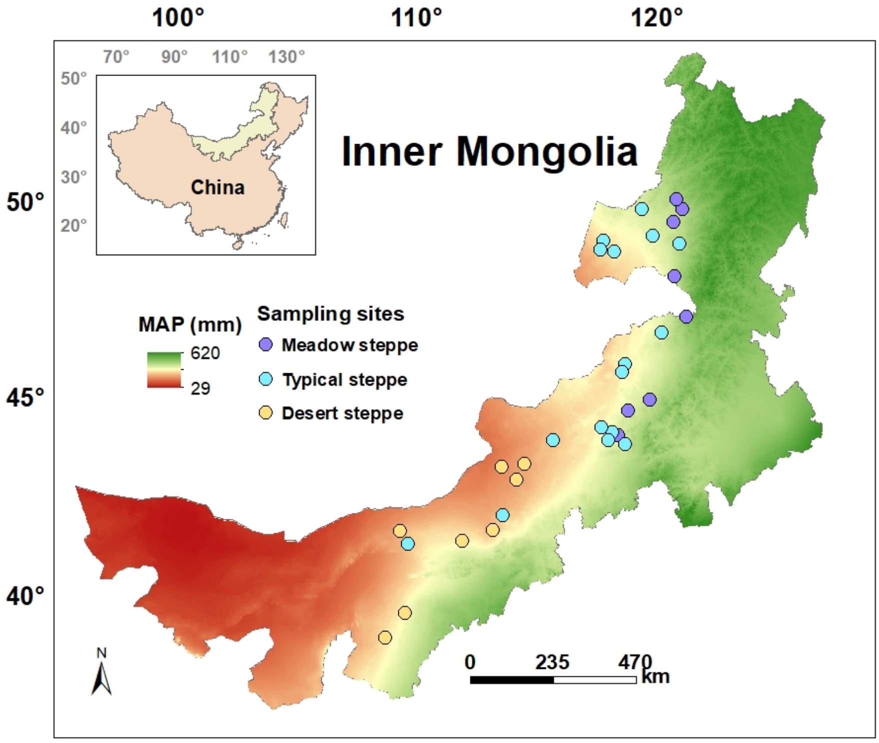

Figure S1. Sampling sites along an aridity gradient in arid and semi-arid grasslands. In total, 32 sites were sampled across desert (yellow dot), typical (blue dot), and meadow (purple dot) steppes from the southwest towards the northeast. MAP−denotes mean annual precipitation in mm, which was obtained from the WorldClim website (http://worldclim.org/version2) [30] in May 10, 2017.
